# Leveraging Diverse Data Sources to Identify and Describe U.S. Health Care Delivery Systems

**DOI:** 10.5334/egems.200

**Published:** 2017-12-15

**Authors:** Genna R. Cohen, David J. Jones, Jessica Heeringa, Kirsten Barrett, Michael F. Furukawa, Dan Miller, Anne Mutti, James D. Reschovsky, Rachel Machta, Stephen M. Shortell, Taressa Fraze, Eugene Rich

**Affiliations:** 1 Mathematica Policy Research, US; 2 Agency for Healthcare Research and Quality, US; 3 University of California, Berkeley School of Public Health, US; 4 The Dartmouth Institute for Health Policy and Clinical Practice, US

**Keywords:** healthcare delivery systems, survey data, federal data, healthcare organizations, physicians, hospitals

## Abstract

Health care delivery systems are a growing presence in the U.S., yet research is hindered by the lack of universally agreed-upon criteria to denote formal systems. A clearer understanding of how to leverage real-world data sources to empirically identify systems is a necessary first step to such policy-relevant research. We draw from our experience in the Agency for Healthcare Research and Quality’s Comparative Health System Performance (CHSP) initiative to assess available data sources to identify and describe systems, including system members (for example, hospitals and physicians) and relationships among the members (for example, hospital ownership of physician groups). We highlight five national data sources that either explicitly track system membership or detail system relationships: (1) American Hospital Association annual survey of hospitals; (2) Healthcare Relational Services Databases; (3) SK&A Healthcare Databases; (4) Provider Enrollment, Chain, and Ownership System; and (5) Internal Revenue Service 990 forms. Each data source has strengths and limitations for identifying and describing systems due to their varied content, linkages across data sources, and data collection methods. In addition, although no single national data source provides a complete picture of U.S. systems and their members, the CHSP initiative will create an early model of how such data can be combined to compensate for their individual limitations. Identifying systems in a way that can be repeated over time and linked to a host of other data sources will support analysis of how different types of organizations deliver health care and, ultimately, comparison of their performance.

## Introduction

Health care delivery systems are a growing presence in the U.S. [1,2,3,4,5] and are part of a national shift from small, independent practices to larger, consolidated organizations [6,7,8,9]. Yet, evidence of the benefits of organized health care delivery systems on value is limited, with some studies suggesting systems may be associated with increased costs and little or no improvements in quality [1,4,5,10,11]. Economic and policy factors have incentivized growth of diverse organizational structures across the spectrum of care delivery. These vertically-integrated health systems have the promise of improving value through learning, care coordination, and increased efficiencies in care delivery [12]. Much of the relevant literature has focused on the organization of hospitals and physician practices; less attention has been given to the systems that integrate physicians and hospitals into the same entity, despite the role these integrated entities may have in determining which health care services are provided. Furthermore, research is hindered because there is no universally agreed-upon definition or set of criteria to differentiate a formal health care delivery system from a more limited hospital-physician partnership or loose network [13,14,15]. Differences in definitions, as well as differences in the methods to specify linkages between physician practices, hospitals, and other settings, present challenges to conducting research and designing policies that promote effective care delivery models or foster more high performing systems.

A clearer understanding of how to leverage real-world data sources to reliably identify and characterize health care delivery systems is a necessary first step to such policy-relevant research. In this paper, we utilize a definition of health care delivery systems developed by the Agency for Healthcare Research and Quality’s (AHRQ) Comparative Health System Performance (CHSP) initiative: systems must include “*at least one general acute care hospital and at least one group of physicians that provides comprehensive care (including primary and specialty care) who are connected with each other and with the hospital through common ownership or joint management* [16].” We use this definition as the basis to explore the following questions:

1. What approaches can be used to identify and describe health care delivery systems using available national data sources?
2. What are the challenges of using these available data sources to identify and describe health care delivery systems?

This paper lays the foundation for future health systems research by providing insights on available data sources to identify and describe health care delivery systems. We aim to stimulate dialogue among researchers about the best ways to use existing data and to map the boundaries of what current data can reveal.

## Background

We draw from our experience in the AHRQ CHSP initiative to select the data sources available to identify and describe health care delivery systems across the US. Launched in 2015 to advance systems research and improve the use of evidence among systems, the CHSP initiative established a Center of Excellence (CoE) at Dartmouth College, the National Bureau of Economic Research, and RAND, as well as a Coordinating Center at Mathematica Policy Research. CHSP seeks to enumerate health care delivery systems and their owned/managed physicians and hospitals to facilitate comparative research on the structural attributes associated with high performance [17,18]. The CoEs are charged with studying, characterizing, and measuring the performance of health care delivery systems as well as identifying the characteristics of high-performing systems and the mechanisms by which systems disseminate and use evidence. The initiative also aims to disseminate resources and findings to advance systems research and inform future data collection efforts.

### Health Care Delivery System Definition

As noted above, CHSP’s definition of a health care delivery system prioritizes vertical integration of “*at least one general acute care hospital and at least one group of physicians*” The definition not only invokes integration across inpatient and outpatient settings but also requires a broad spectrum of care delivered (“…*provides comprehensive care (including primary and specialty care*”). The definition requires a formal relationship among these diverse providers (“…*connected with each other and with the hospital through common ownership or joint management*”) but does not require any particular mechanism for formalizing these relationships. For example, a hospital that employed community-based physicians who provide comprehensive care (but were not organized as an independent medical group) would be considered a health care delivery system. Additionally, foundation models are considered a form of joint management; however, in the CHSP definition, joint contracting with payers – such as through a Physician Hospital Organization (PHO) or an Accountable Care Organization (ACO) – is not itself indicative of joint management. Likewise, many other provider organization relationships prominent in the U.S. today would not meet the CHSP definition of a health care delivery system because they do not span primary and specialty care in both the outpatient and inpatient setting, including horizontally integrated systems such as multi-specialty group practices, multi-hospital chains or multiple physician practices contracting with payers through an Independent Practice Association.

### Health Care Delivery System Elements and Data Needs

The task of identifying and describing health care delivery systems is itself a complex undertaking. Available secondary data sources and the data elements they contain further create boundaries on researchers’ abilities to implement and operationalize these definitions. Thus, researchers must first specify the data elements that are needed for their definition of health care delivery systems using available secondary data sources. These elements include variables that describe provider entities (for example, hospitals and physician practices) and health care services; identify affiliations or relationships among the provider entities (for example, system ownership or contractual relationships); and, have uniform identifiers across data sources so researchers can attribute individual providers to systems, identify additional health system attributes, and measure health system performance.

**Figure 1:**
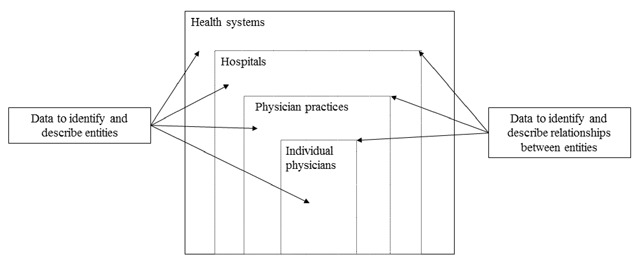
Data Needs to Study Vertically Integrated Systems.

In light of the multi-layered and heterogeneous composition of vertically integrated health care delivery systems, various data sources are needed to identify system owners as well as link providers and organizations that are part of those systems. Figure 1 illustrates the organizational and professional relationships that lie within each health care delivery system, underscoring the volume of data (typically from multiple data sources) needed to fully understand and characterize vertically integrated systems.

### Data Sources

As part of CHSP, AHRQ, the three CoEs, and the Coordinating Center identified national data sources that can be used to identify and describe health care delivery systems. While there are a host of data sources that are useful in studying system components and their attributes, such as the American Medical Association (AMA) Masterfile and Medicare Data on Provider Practice and Specialty (MD-PPAS), only a handful explicitly identify systems or indicate relationships among organizations that can be used to identify which collections of organizations constitute a system. These data sources are:

- American Hospital Association (AHA) annual survey of hospitals
- Healthcare Relational Services (HCRS) Databases, including the Healthcare Organization Services (HCOS) database and the Healthcare Professional Services (HCPS) database
- SK&A Healthcare Databases
- Provider Enrollment, Chain, and Ownership System (PECOS)
- Internal Revenue Service (IRS) 990 forms

We describe the data sources in detail, focusing on their provenance, utility for identifying health care delivery systems, and strengths and limitations. We then discuss the process of incorporating multiple sources to study systems, including unique identifiers that will enable both the identification of systems and the means with which to link organizations across data sources.

#### AHA Survey

The AHA fields a serial cross-sectional survey of the over 6,400 U.S. hospitals operating in the U.S.; the survey typically has a response rate over 75 percent [19]. The objective of the survey is to track and monitor the evolution of new systems of care, care coordination functions, and the various payment models providing care to a population as they are experienced by hospitals. Data are supplied by hospital administrators online, as well as by paper and pencil. The AHA survey is available for purchase (see Table 1).

**Table 1:** Key Data Sources for Health System Identification.

Source	Data Holder	Data Collection and Purpose	System Identifiers	System Components	Availability

**America Hospital Association (AHA) Annual Survey**	AHA	Membership database for policy research and industry monitoring	Common ownership among providers and organizations	Hospitals owned by systems	Available for purchase under a variety of pricing schemes based on the options selected
**Healthcare Relational Services (HCRS)**	IQVIA (formerly Quintiles IMS)	Reference database for sales/marketing purposes	Integrated Health Systems	Providers/hospitals owned by hospitals and systems	Available for purchase under a variety of pricing schemes based on the options selected
**SK&A Healthcare Databases**	IQVIA (formerly Quintiles IMS/SK&A)	Reference database for sales/marketing purposes	Integrated Healthcare Delivery Networks (IDNs)	Providers/hospitals and their ownership	Available for purchase under a variety of pricing schemes based on the options selected
**Medicare Provider Enrollment, Chain, and Ownership System (PECOS)**	Centers for Medicare and Medicaid Services (CMS)	Enrollment database for the purpose of Medicare program participation	Vertical integration among associated organizations	Chain home office and owner/manager reported for providers/hospitals	Public for a limited version, through DUA for the full version
**Internal Revenue Service (IRS) 990 and Schedule R Forms**	IRS	Financial database for the purpose of tax reporting	Vertical integration among associated organizations	Related organizations under not-for-profit systems	Publicly available

The AHA survey provides several mechanisms for identifying health care delivery systems. The data may be used to identify horizontally integrated hospitals, identify hospitals which have vertical relationships with physicians, and characterize the nature of these relationships in a health system taxonomy. The relationships include hospital ownership of physicians through integrated salary models or equity models; physician group or physician ownership of hospitals; hospital participation in foundation models; and hospital-physician alignment through management services organizations and PHOs (see Table 2). Researchers studying vertical integration have commonly used the data to examine hospital arrangements with physicians. The AHA survey collects data on 8 different types of arrangements, three of which represent ownership relationships of physicians and denote vertical integration (foundation models, integrated salary models, or equity models) [11]. In addition, the AHA survey can be used to employ a health system taxonomy that assigns hospitals into one of five different types of systems based on their level of differentiation, centralization, and integration [20,21,22]. The survey also provides a system membership variable which tracks hospitals’ membership in a diversified single hospital or multihospital health care system, defined as “two or more hospitals owned, leased, sponsored, or contract managed by a central organization.” These constructs are not mutually exclusive and the latter two have an emphasis on horizontal as opposed to vertical integration. In 2013 the AHA also began fielding a supplemental Survey of Care Systems and Payment designed to track and monitor the evolution of new systems of care, including ACOs and other entities engaged in various payment models. Survey questions cover hospitals’ activities with care coordination and data sharing as well as participation in financial risk arrangements such as bundled payment and shared savings [23,24].

**Table 2:** Data elements for defining health systems in key national secondary data sources.

	AHA	HCRS	SK&A	PECOS	IRS 990

**System entities**	Hospitals that are AHA members	Hospitals Single and multispecialty group practices Nursing homes Individual clinicians Other providers	Hospitals Physician practices Nursing homes Individual clinicians	Hospitals Medicare-enrolled physician group practices Medicare-enrolled individuals clinicians	Certain nonprofit health care organizations including hospitals and health systems
**Provider specialty and health care services information**	Hospital type Specialty of physicians with hospital privileges Total number of affiliated physicians^1^	Physician specialty Hospital type Total number of affiliated physicians	Physician specialty Hospital type Total number of affiliated physicians	Physician specialty Total number of affiliated physicians	Number of hospital facilities Type of hospital (e.g., children’s, teaching)
**Relationships**	Diversified single or multihospital systems Hospital-physician arrangements^2^	Ownership, management, co-business, leasing, or purchasing affiliations among corporate entities or integrated delivery networks and distinct health care organizations^3^	Ownership or management of groups, hospitals, and other health care facilities by a common corporate entity	Ownership and management control associations tied to organizational enrollment^4^	Entities must report on any relationships that are considered partnerships with “related” organizations^5^ Entities must also report on any “unrelated” organizations with which they conducted more than 5 percent of its activities^6^
**Linkage variables**	American Hospital Association (AHA) ID CMS Certification Number (CCN)	National Provider Identifier (NPI) CCN	NPI AHA ID	NPI CCN Tax Identification Number (TIN)	TIN

Notes: This table focuses on data elements and system components that are relevant to identifying and describing health systems. Data sources may contain information about other entities, such as durable medical equipment suppliers, that are not presented here. The meaning of the data elements identifying relationships and affiliations among system entities are nuanced and data source-specific; researchers need to fully understand these elements and may need to use multiple elements in concert to identify the relationships and establish hierarchies among the various entities (for example, assigning parent hospitals to system owners). The relationships described here are high-level, intended to indicate the nature of the relationships represented in the data. AHA and HCRS provide the total number of affiliated physicians, while PECOS and SK&A provide the data to enable researchers to calculate this element. Includes hospital ownership of physicians through integrated salary models or equity models; physician group or physician ownership of hospitals; hospital participation in foundation models; and hospital-physician alignment through management services organizations and physician-hospital organizations. Relationships in HCRS includes five relationship categories (ownership, purchasing distribution, co-business, and academic) and 37 types of relationships, including pharmacy and medical/surgical purchasing and distribution affiliates. These relationships indicate the type of entity as well as the nature of the relationship and may be used in concert with other variables that indicate the hierarchical relationships among system entities. Relationships or enrollment associations in PECOS span 34 categories; only a subset are directly relevant to identifying health systems. Includes parent, subsidiary, brother/sister, or supporting/supported organizations. Activities are based on total assets or total revenue for the tax year. Sources: Analysis of provider relationships and enrollment associations in the HCRS 2016 data and PECOS data as of August 2016. IMS Health. Healthcare Organization Services: Professional and organization affiliations maintenance process. Bedford, NH: IMS Health. Available at: http://us.imshealth.com/legal/ServicePlanDetails-HCOS.pdf.; American Hospital Association. 2015 AHA Annual Survey Health Forum. Chicago, IL: AHA. Available at: https://www.ahadataviewer.com/Global/survey%20instruments/2015AHAAnnualsurvey.pdf.; Centers for Medicare & Medicaid Services. PECOS for provider and supplier organizations. Baltimore, MD: CMS; 2016. Available at: https://www.cms.gov/Outreach-and-Education/Medicare-Learning-Network-MLN/MLNProducts/downloads/MedEnroll_PECOS_ProviderSup_FactSheet_ICN903767.pdf.; Internal Revenue Service. Instructions for Schedule R (Form 990). Washington, D.C.: IRS; 2015. Available at: https://www.irs.gov/pub/irs-pdf/i990sr.pdf. ; Internal Revenue Service. Schedule H (Form 990). Washington, D.C.: IRS; 2016. Available at: https://www.irs.gov/pub/irs-pdf/f990sh.pdf; SK&A. Integrated Health Systems Data. 2017. Available at: http://www.skainfo.com/databases/integrated-health-systems.

The AHA survey is commonly used in health services research literature and contains a host of other relevant measures that might be useful in studying health system attributes. For example, the AHA survey collects detailed information on hospital capacity, finance, and use of health information technology (HIT). Furthermore, although the survey has a cross-sectional design, AHA ID can be used for cohort studies to monitor changes in hospital behavior over time [25]. A chief limitation of the AHA survey for the purposes of understanding health care delivery systems is its limited data on physician practices or providers. For example, while the AHA describes hospitals’ relationships with physicians, it does so at a summary level for physicians and practices without providing additional details on those entities.

#### Healthcare Relational Services (HCRS)

IQVIA (formerly Quintiles IMS) maintains two integrated databases relevant to the study of health system performance under the umbrella of Healthcare Relational Services (HCRS). The first, the Healthcare Organization Services (HCOS) database, focuses on health care organizations. The second, the Healthcare Professional Services (HCPS) database, focuses on the health professionals that work in those organizations (e.g., physicians and physician extenders, such as nurses and physician assistants). Information contained within HCOS and HCPS is updated regularly based on manual web searches, telephone verification, and information received from the AMA, National Plan and Provider Enumeration System (NPPES), the Drug Enforcement Agency (DEA) registration files, state licensing agencies, and drug distribution data non-retail shipping addresses. HCRS data are available for purchase under a variety of pricing schemes based on the options selected (See Table 1).

Of the 8.1 million professionals in HCPS, approximately 2.5 million are affiliated with organizations contained within HCOS. Linking professionals to organizations via location is done with proprietary address intelligence software and matching algorithms. HCOS contains information on approximately 650,000 medical group practices, hospitals, ACOs and other organizations, including organizational characteristics such as bed count, provider counts, HIT, and finances. HCRS specifies five relationship categories (ownership, purchasing, distribution, co-business, and academic) and 37 types of relationships, including pharmacy and medical/surgical purchasing and distribution affiliates. These relationships indicate the type of entity as well as the nature of the relationship and may be used in concert with other variables that indicate the hierarchical relationships among system entities. Within HCOS, systems are defined as Integrated Healthcare Delivery Networks (IDNs), health care organizations that have “direct responsibility for centralizing the purchasing or contracting of its affiliated hospitals and ancillary-care facilities” that also “offer a continuum of care through services at acute and non-acute sites” (See Table 2) [26].

HCRS has many advantages for health care delivery systems research, particularly because it offers multiple databases that can be related to each other. For example, providers in the HCPS database can be associated with organizations in the HCOS database. However, there is a limited ability to link HCRS organizations to organizations in other data sources due to lack of tax identification numbers (TINs).

#### SK&A

IQVIA also maintains the SK&A databases. SK&A is a private “provider of U.S. health care reference information” that creates lists of physicians, hospitals, ACOs, and other health care providers [27]. The physician database specifically profiles physicians and other office-based prescribers, including residents. SK&A processes data from DEA registration files, National Provider Identifier (NPI) files, and company and corporate directories to identify existing providers. Information is validated through phone calls to practice site locations every six months, and the database is updated on a rolling basis. SK&A databases are primarily designed to support marketing by providing information about medical groups, but are increasingly used for research purposes. The data are available for purchase under a variety of pricing schemes based on the options selected (see Table 1).

SK&A defines an “integrated health system” based on common ownership or management of groups, hospitals, and other health care facilities by a common corporate entity. SK&A uses both a bottom-up and top-down approach for identifying health systems. For the bottom-up approach, SK&A calls physician practice sites and inquires about the ownership or management of the practice site. This approach is augmented by a top-down approach wherein SK&A searches health system web sites, trade publications and other sources for health system information. The physician database includes a number of descriptive variables, such as location, size, and HIT adoption and use (See Table 2).

SK&A databases provide a wide range of information on different organizations and individuals providing health care. It has many advantages for health systems research, particularly because it offers multiple databases that can be related to each other. For example, each physician in the physician database can be associated with a practice and a hospital. Both physician groups in the physician database and hospitals in the hospital database can be associated with systems and ACOs. However, data only include office-based physicians. Furthermore, there is potential measurement error because information is provided through survey responses given by physician office staff.

#### PECOS

PECOS is a national data repository for individual and organizational providers enrolled in Medicare. This electronic database enables physicians, non-physician practitioners, and provider and supplier organizations to enroll in Medicare, make a change in their Medicare enrollment, view their Medicare enrollment information on file with Medicare, or check on the status of a Medicare enrollment application. PECOS includes a small number of enrollment forms that are not processed electronically, chiefly for enrolling providers that do not wish to submit information online. The data are maintained by the Centers for Medicare & Medicaid Services (CMS) and are updated quarterly. A limited set of PECOS variables are publicly available, but most data may only be accessed by researchers who are working on government contracts or with approval from CMS through the Research Data Assistance Center. Researchers may gain access to one of several versions of PECOS, including the “Global Extract,” which is produced quarterly (See Table 1).

The Global Extract includes information on provider participation status and practice location information [28]. In PECOS, providers must indicate whether they are sole owners of professional association, professional corporation, or limited liability corporation; self-employed; a group member only; a group member and self-employed; or a disregarded entity. Relationships or enrollment associations in PECOS span 34 categories; only a subset are directly relevant to identifying health systems. PECOS identifies providers using NPI and identifies organizations by TINs. While the data do not report an overarching system or parent entity, it is possible to construct a network of related entities using linkages between NPIs and TINs and associations listed across TINs. For example, TINs for a set of physician groups and hospitals enrolled in PECOS might be listed as having associations with each other and a chain home office that is a vertically integrated organization (See Table 2).

There are inherent challenges in repurposing administrative data for research, such as the fact that variables like organization size must be manually calculated by counting the number of physicians who report practicing at the same practice TIN and address. This process is challenged by the way physicians are associated with TINs, such that some physicians may practice together under different TINs whereas other physicians may practice separately while sharing the same TIN. Moreover, the PECOS data are restricted to the subset of physicians enrolled in Medicare, which may exclude some providers such as pediatricians. However, because PECOS enrollment is a prerequisite to receiving payment from Medicare, there is a strong incentive for providers who are enrolled to maintain accurate information, which could provide more accurate information when compared to data collected through surveys fielded by third-party organizations.

#### IRS 990 Forms

IRS 990 forms include information that health care organizations exempt from paying income tax (such as certain non-profit health care organizations) submit annually to the IRS. Tax-exempt organizations that report relationships with other organizations and/or entities are required to file Schedule R, Related Organizations and Unrelated Partnerships, forms. Electronically submitted IRS 990 forms and schedules are available through Amazon Web Services (AWS), free of charge (see Table 1).

IRS 990 forms contain information about the submitting organizations, including their activities, governance, and revenue. Schedule R forms document the name and TIN of organizations with which they have a relationship. Depending on the nature of the relationship, information about each organization’s share of total income, share of assets, and percentage ownership is provided by the submitting organization. The IRS 990 forms and Schedule R forms do not indicate if an organization is a health system, but form 990 data can be used to link entities that have relationships with non-profit tax exempt hospitals using Schedule R, on which organizations list related organizations and partnerships, including their TINs (see Table 2). Relationships that are considered partnerships with “related” organizations includes parent, subsidiary, brother/sister, or supporting/supported organizations.

IRS variables related to finances and relationships, such as Medicare revenue and community health needs assessments, may be useful for providing a context for understanding research on health system performance. However, because of the structure of the files, accessing a full year of IRS 990 and Schedule R returns is time-consuming and requires substantial programming expertise and resources to convert the data to a usable database format. In addition, the forms are only available for tax-exempt organizations; private, for-profit organizations (such as investor owned hospitals as well as diverse physician practice organizations) are not included. Furthermore, only about 25 percent of the roughly 1.6 million non-profit organizations are required to file the IRS 990 and, of these, only forms filed electronically and can be accessed via AWS. This could limit the generalizability of conclusions drawn from IRS data and also introduce measurement error into analyses featuring these data by excluding organizations with lesser technical capabilities. Despite these limitations, since these reports to the IRS are required to maintain tax exempt status, the content of these reports is guided by IRS regulations, and the information is reported by the organizations themselves (rather than collected by a third party), it is likely the information reported is reasonably current and accurate [29,30,31,32].

### Incorporating Multiple Data Sources to Study Health Care Delivery Systems

The highlighted data sources enable researchers to study how vertically integrated health systems in the U.S. deliver patient care. At the highest level, these data capture organizations that have shared ownership or joint management relationships among system components that provide health care services. Each data source has its strengths and limitations for studying and describing health care delivery systems. For example, federal sources like PECOS and IRS are almost perfectly complete for a specific subset of health care organizations, such as Medicare providers and non-profit providers, respectively. In contrast, private sources like AHA, HCRS, and SK&A have lower response rates, and thus more missing information, for a broader population of organizations. Similarly, research employing these data sources will inevitably be influenced by the original purposes of the data and the ways in which the data were collected. For example customers of SK&A data include pharmaceutical companies attempting to market prescription medications to ambulatory care physicians; therefore practice location and clinical focus of these physicians is likely to be accurate, but hospital-based physicians may not be consistently observed [33].

Scholars have also compared the benefits of different data sources, including enrollment and survey data, identifying some tradeoffs. Notably, one study found that the proportion of physicians practicing in large practices was substantially higher in federal administrative data than in national survey data [34]. Another study observed that while three common sources of physician data offer mostly correct and complete information, their relative utility varies by specialty and some physicians only appear in one or two of the three sources [33]. Finally, scholars compared the different patterns of hospital ownership of physician practices that emerge when using hospital- vs. physician-focused surveys, noting the importance of surveying both entities to fully understand trends [20]. These lessons highlight the complexity and nuances when interpreting data about health care providers and how they organize themselves.

As an initial step, the CHSP initiative is examining vertically integrated systems characterized by shared ownership or management that are defined in AHA, HCRS, and SK&A data. The initiative will compare the results from these data sources with data reported through federal data sources such as PECOS and the IRS form 990 data with the goal of producing lists of vertically integrated health systems and their affiliated hospitals, physician practices and physicians. These systems identified through federal data sources can be used to augment and corroborate the information reported in other sources, such as AHA, HCRS, and SK&A. In addition to providing added context regarding the specific relationships among systems’ members, the data sources have the advantage of being provided directly from the providers themselves which adds an additional level of completeness and validation to systems and relationships developed using the data.

Incorporating these additional data sources opens a wealth of opportunities to examine whether and how features of systems impact the quality and cost of care by solidifying the detailed relationships among members of systems that can be linked to other data sources. For instance, identification and description of health systems would facilitate qualitative studies of learning health systems and also provide sampling frames for surveys that directly examine learning health system and their impact on system performance [35].

**Figure 2:**
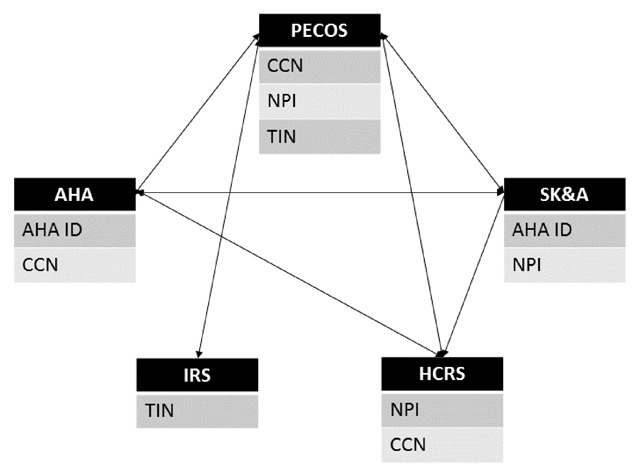
Data Source Linkages. Data Source Acronyms, clockwise from top: Provider Enrollment, Chain, and Ownership System (PECOS); SK&A Healthcare Databases (SK&A); Healthcare Relational Services Databases (HCRS); Internal Revenue Service 990 forms (IRS); American Hospital Association annual survey of hospitals (AHA). Matching Variable Acronyms, alphabetically: American Hospital Association ID (AHA ID); CMS Certification Number (CCN); National Provider Identifier (NPI); Tax Identification Number (TIN). Notes: Data sources specified in the black boxes may be linked through the matching variables specified in the gray boxes.

Though linking data sources can be challenging, it is possible to combine them. There is no identifier that can be used to link systems across all data sources, but one can use systems’ names and reported relationships to identify the same entities across data sources. For example, system names are fairly consistent in AHA, HCRS, and SK&A. Furthermore, the sources typically contain a very similar set of hospitals, which can be used to link systems across sources by hospital identifiers. Specifically, AHA has AHA IDs, which the AHA assigns to all member hospitals, and the CMS Certification Number (CCN), which CMS assigns to all facilities participating in Medicare. SK&A has AHA IDs and HCRS has CCNs. Thus, even if all of the systems’ names can’t be matched in an automated fashion between these two sources, they can be linked through the hospital identifiers. In addition, multiple data sources contain TINs, which the IRS assigns to all organizations, and NPIs, which the CMS NPPES assigns to all HIPAA-covered providers and providers who bill for Medicare services. These identifiers can be used to link physician practices and physicians, respectively (see Figure 2).

Through our work on the CHSP initiative, we find that the AHA, HCRS, and SK&A data largely contain the same set of component entities; however, they define systems differently, which leads to a different set of systems and associations between entities and systems. This occurrence is particularly true for hospitals and their relationships captured within systems. For example, two sources may disagree on whether or not a hospital is part of a system based on different requirements regarding ownership or the continuum of care provided by the system. Such differences might occur because of nuances in how the various sources view or define systems. Differences can also occur due to response errors when respondents are used to characterize systems. For instance, HCRS defines IDNs, which could include organizations that have relationships with their component entities that do not qualify as ownership or management; whereas the integrated health systems listed in the SK&A data must have ownership or management relationships with their component entities. Similarly, one source might not list a smaller local system that is part of a larger multi-regional or national system as a system, while another source lists both as related systems in a nested relationship.

Determining the best way to use the data sources in concert with one another depends on how the researcher wants to define health care delivery systems. For example, the CHSP initiative aims to identify vertically integrated systems providing comprehensive care. The information available to inform study design decisions includes the nesting of the organizations (such as the physicians in practices, practices in hospitals, and hospitals in systems); the relationship types (ownership, management, or a looser affiliation); and characteristics of the component entities (the number and types of physicians and hospitals). Taken together, the data sources highlighted in this paper have fairly comprehensive information to inform decisions regarding how to define systems to meet a broad range of research objectives. For example, investigators may be interested in examining the relationship between various types of hospital-physician system linkages and ambulatory care sensitive admission rates or 30 day unplanned hospital readmissions. The CHSP data sources can also be linked to primary data collection survey efforts to assess, for example, what types of systems, hospitals and physician practices are furthest along in adopting and implementing biomedical, care management, patient engagement and related innovations.

## Discussion

No single national data source provides a complete picture of U.S. health care delivery systems and their component hospitals, physician practices and physicians, particularly in light of frequent mergers, acquisitions, and other organizational changes. But, combining various sources can help compensate for each source’s limitations. Moreover, when the information overlaps across sources, it can be used for validation. AHA, HCRS, and SK&A data provide similar information on high-level constructs of systems and the hospitals and practices linked to these systems. Federal data sources provide an additional source of information to validate the relationships captured in the AHA, HCRS, and SK&A data, which could be particularly illuminating because the data on linkages are reported directly by the entities comprising systems.

Sources that specify relationships between entities provide a promising opportunity to construct horizontally and vertically integrated systems from the ground up using information reported directly by those providers and organizations delivering care. Being able to examine linkages among system components gives researchers the flexibility to tailor the definition of systems according to the objectives of their analyses. For example, when data contain characteristics of the entities being linked (such as the number and types of physicians and their relationships with hospitals), researchers may apply additional criteria for size and scope that they deem important to understanding health system performance. Moreover, having common identifiers for hospitals (AHA IDs and CCNs), physician practices (TINs), and physicians (NPIs) enables researchers to link data sources with explicit definitions of health systems to these health systems that were identified from the ground up, further enabling researchers to fill in the details of characteristics of the systems’ component entities and the systems themselves.

In addition to the data sources discussed here, there are many other sources available that can be linked to health care delivery system information to add to understanding of how care is organized and delivered. Claims for health care services (Medicare, Medicaid, and commercial) have been linked to physicians and physician practices to provide information on services provided, referral patterns, quality metrics, and costs of care. Indeed, a recent article articulated how NPIs can be linked to Medicare and Medicaid fee-for-service claims as well as the AMA Masterfile for research purposes [36,37,38]. Furthermore, in a few efforts, claims data have been linked to electronic health record data to provide more clinically nuanced information on patient conditions and care processes [39]. Similarly, quality and cost data from public sources such as Physician Compare and Hospital Compare can be linked to other data sources on physicians and hospitals. In addition, there are survey data available at the national and regional levels (for example, the National Survey of Small and Medium-Sized Physician Practices and Medical Group Management Association survey) containing information that can be linked to subsets of physicians, physician practices, and hospitals, including direct reports of relationships with health care delivery systems [40,41]. State and regional sources that identify local health care delivery systems and linkages are also increasingly available to researchers (for example, the Massachusetts Registry of Provider Organizations), which can be used to supplement and validate the information discussed here [42]. Moreover, there are a number of data sources from the federal government, private vendors, and collected by research teams that identify and describe ACOs and their relationships to vertically integrated systems [43,44,45,46,47]. Finally, CMS has indicated it will report provider participation in diverse Medicare alternative payment models (APM) including various new forms of Medicare ACOs. These data can be used to identify and characterize provider relationships to explore these alternative constructs of health care delivery systems contrasted with the formal vertically integrated systems highlighted in this paper.

The information presented in this paper illustrates some of the challenges in using the currently available national data and linking variables for the component hospitals, physician practices, and physicians to describe the structure of health care delivery system. We note some options for how these data might be used and how such identification of health care delivery systems can support analysis of how these entities organize their delivery of care. The CHSP initiative recently released an inaugural Compendium of U.S. Health Systems, which provides an early model of how such data can be used to identify systems in a way that can be updated and repeated over time and linked to a host of other data sources to support analysis of how different types of organizations deliver health care [16]. There are tremendous possibilities for this line of research using data sources to identify organized health care delivery systems.
